# Takotsubo Syndrome Following Status Epilepticus in a Heart Transplant Recipient: A Case Report

**DOI:** 10.5811/cpcem.41970

**Published:** 2025-01-17

**Authors:** Takeshi Shikama, Mio Shikama, Naoki Hayase, Kent Doi

**Affiliations:** The University of Tokyo, Department of Emergency and Critical Care Medicine, Tokyo, Japan

**Keywords:** Takotsubo syndrome, heart transplantation, reinnervation, case report

## Abstract

**Introduction:**

Takotsubo syndrome (TTS) expresses transient wall motion abnormality of the left ventricle, reportedly induced by sympathetic overstimulation. Takotsubo syndrome is unlikely to be included in the differential diagnosis of heart transplant patients with sudden cardiac dysfunction given the complete denervation occurring during the transplantation.

**Case Report:**

In this case report we describe the case of a female heart transplant recipient who showed apical ballooning on an echocardiogram following status epilepticus. Detailed clinical examinations and her clinical course confirmed the diagnosis of TTS. An iodine-123 meta iodobenzylguanidine myocardial scintigraphy revealed partial cardiac sympathetic reinnervation in the transplanted heart.

**Conclusion:**

This case demonstrates that TTS can manifest itself even in a transplanted heart with partial sympathetic reinnervation.

## INTRODUCTION

Takotsubo syndrome (TTS) is characterized by a reversible left ventricular dysfunction that commonly represents hypokinesis of the apical segment of the left ventricle (LV) after emotional and physical stress.^1^ It is believed that sympathetic overstimulation may play an important role in TTS pathogenesis. In theory the autonomic nerve signals in the transplanted heart should be blocked since allografts are entirely denervated during heart transplantation.^2^ Here, we present a case of a female patient who developed TTS following status epilepticus 15 months after heart transplantation.

## CASE REPORT

A 44-year-old woman, diagnosed at age five with hypertrophic obstructive cardiomyopathy, had undergone heart transplantation for the condition 15 months prior to her transfer to our emergency department (ED) after three witnessed episodes of seizure lasting a few minutes. Altered mental status on the scene was observed prior to the arrival of paramedics who found her unresponsive with rightward conjugate eye deviation.

Two years before the transplant surgery, a continuous-flow left ventricular assist device had been implanted as a bridge to the transplantation because of severely worsening heart failure. She also suffered from a cardiogenic cerebral embolism, infectious intracranial aneurysm, and symptomatic epilepsy secondary to those cerebrovascular complications. After successful heart transplant surgery, she’d had no severe complications since then. Her immunosuppressants contained tacrolimus and mycophenolate mofetil. Additionally, her antiepileptic regimen included levetiracetam, zonisamide, and lacosamide. She took these antiepileptics regularly. However, dose adjustment was frequently required because of several episodes of epileptic seizures in the most recent six months.

On ED arrival, the patient manifested generalized tonic seizures with a Glasgow Coma Scale score of E4V1M1. Her blood pressure was 140/100 millimeters of mercury, heart rate 135 beats per minute, respiratory rate 15 breaths per minute, and oxygen saturation 99% under oxygen supplementation by mask with reservoir at 15 liters per minute. The convulsion was terminated immediately after 10 milligrams (mg) diazepam was administered intravenously (IV). Additionally, she received 1000 mg IV levetiracetam.

**Image 1:**
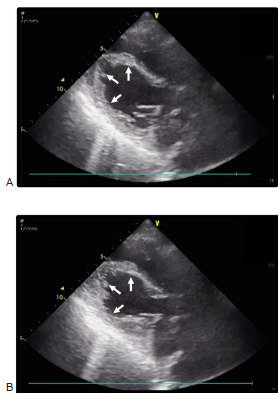
Long-axis achocardiogram view at end-diastole (A) and end systole (B) on admission, showing apical ballooning (white arrows) in the left ventricle.

Because we suspected cardiac syncope due to acute coronary syndrome, we measured cardiac enzymes, which revealed the following: high-sensitivity cardiac troponin I, 1,872 picograms per milliter (pg/mL) (reference range: 0–26.2 pg/mL); creatine phosphokinase, 109 units per liter (U/L) (43–165 U/L); creatine phosphokinase-Muscle/Brain, 10 U/L (0–15 U/L). A chest radiograph showed no pulmonary edema. Electrocardiogram revealed a regular sinus rhythm without significant changes in the ST segment. Transthoracic echocardiogram demonstrated hypokinesis of the left ventricle (LV) apical segment, which extended beyond the territory of a single coronary artery (Image 1). Computed tomography and magnetic resonance imaging of the head showed an embolized aneurysm of the right middle cerebral artery (MCA) and an old infarction in both MCA areas, but no acute lesions.

**CPC-EM Capsule**

What do we already know about this clinical entity?

*Takotsubo syndrome (TTS) expresses transient wall motion abnormality of the left ventricle. The possible pathogenetic mechanism is sympathetic overstimulation*.

What makes this presentation of disease reportable?

*In this case, TTS manifested in a transplanted heart with partial sympathetic reinnervation*.

What is the major learning point?

*TTS can develop in a transplanted heart despite sympathetic reinnervation occurring only partially*.

How might this improve emergency medicine practice?

*This case highlights the importance of cardiological evaluation for transplant patients with seizure and of close communication with transplant physicians*.

**Image 2:**
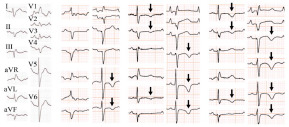
Serial electrocardiogram on day of admission, and Days 4, 10, and 19. Black arrows denote T-wave inversion.

**Image 3:**
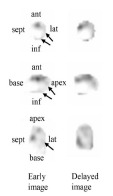
Iodine-123 meta iodobenzylguanidine (MIBG) single-photon emission computed tomography images on Day 34. Black arrows show decreased MIBG accumulation. *ant*, anterior; *lat*, lateral; *inf*, inferior; *sept*, septal.

Based on these findings, systemic tonic convulsions and impaired consciousness were diagnosed as symptomatic epilepsy associated with old cerebral infarction. Because of a prolonged coma, she was admitted to our intensive care unit after endotracheal intubation and sedation with propofol for suspected non-convulsive status epilepticus. Two days later, her consciousness level improved by increasing zonisamide dosage, and she was extubated. T-wave inversion and QTc prolongation were detected on the follow-up electrocardiogram on Day 4 (Image 2). Coronary angiography was electively conducted on Day 13 and showed no findings of obstructive coronary artery disease. Simultaneously, a myocardial biopsy was performed, and no acute cellular or antibody-mediated graft rejection was shown (International Society for Heart and Lung Transplantation Grade 0 and pathologic Antibody-Mediated Rejection Grade 0).^3^,^4^ The wall motion in her LV gradually improved and returned to normal on day 19. The coefficient of variation of R-R intervals (CVRR) was examined on day 20 to evaluate parasympathetic function. We discovered that CVRR was decreased (1.08%). Furthermore, an iodine-123 meta iodobenzylguanidine (^123^I-MIBG) scintigraphy was conducted on day 34. Early and delayed heart-to-mediastinum (H/M) ratios were 1.56 and 1.25, respectively. Global washout rate for ^123^I-MIBG was 39%. Early images of single-photon emission computed tomography showed decreased MIBG accumulation at the inferior-posterior and lateral walls and apex (Image 3). These nuclear medicine evaluations implied that the heart was largely but not completely denervated. Consequently, she was discharged from our hospital on day 38. Per the diagnostic criteria in the International Expert Consensus Document on Takotsubo Syndrome, the reversible wall motion abnormality at the apex of this patient was finally diagnosed as TTS triggered by status epilepticus.^1^

## DISCUSSION

Takotsubo syndrome is a transient LV dysfunction, typically involving the apical segment, without evidence of obstructive coronary artery disease. Although the pathogenetic mechanism is still unconfirmed, there has been emerging evidence that sympathetic overstimulation may play a crucial role in the pathogenesis.^2^ Furthermore, catecholamine-induced toxicity on cardiomyocytes,^5^ microcirculatory dysfunction,^6^ and epicardial spasm^7^ have been identified as potential mechanisms by which excess catecholamine induces myocardial stunning. Recent case reports have shown that TTS occurs in transplanted hearts, although complete allograft denervation commonly occurs during heart transplantation.^8^–^10^ A previous study demonstrated that partial sympathetic reinnervation occurred in up to 40% of recipients one year after heart transplant surgery.^11^ However, some case reports on TTS in transplanted hearts showed no evidence of reinnervation.^9^,^10^

In this case, the result of ^123^I-MIBG scintigraphy presented decreased H/M ratio on both the early and delayed images. Furthermore, MIBG accumulation was reduced at the inferior-posterior and lateral walls and apex. These results indicated that her heart remained largely denervated. However, the anteroseptal area showed myocardial uptake of ^123^I-MIBG, suggesting partial sympathetic reinnervation in her transplanted heart. There are two explanations as to why this patient developed TTS without global sympathetic reinnervation. First, the transplanted heart may be hypersensitive to catecholamines because of the upregulation of β-adrenergic receptors.^12^ Second, insufficient parasympathetic reinnervation corroborated by decreased CVRR may make the heart more susceptible to circulating catecholamines.

Based on this case, we suggest that emergency physicians consider the following points. First, cardiological evaluation is necessary for the ED patient manifesting seizure. Second, emergency physicians should be in close communication with transplant physicians regarding transplant cases presenting to ED because of their complex anatomy and physiology. We were able to diagnose this case in collaboration with transplant physicians.

## CONCLUSION

We present a case of a female patient who developed TTS following status epilepticus 15 months after undergoing heart transplant surgery. This case shows that TTS could develop in a transplanted heart after status epilepticus despite sympathetic reinnervation occurring only partially.
